# Model-based analysis of multi-UAV path planning for surveying postdisaster building damage

**DOI:** 10.1038/s41598-021-97804-4

**Published:** 2021-09-20

**Authors:** Ryosuke Nagasawa, Erick Mas, Luis Moya, Shunichi Koshimura

**Affiliations:** 1 Mox-Motion, Tokyo, 161-0033 Japan; 2 International Research Institute of Disaster Science, Tohoku University, Sendai, 980-8572 Japan; 3 Japan-Peru Center for Earthquake Engineering and Disaster Mitigation (CISMID), National University of Engineering, Lima, Peru

**Keywords:** Environmental sciences, Natural hazards, Engineering

## Abstract

Emergency responders require accurate and comprehensive data to make informed decisions. Moreover, the data should be acquired and analyzed swiftly to ensure an efficient response. One of the tasks at hand post-disaster is damage assessment within the impacted areas. In particular, building damage should be assessed to account for possible casualties, and displaced populations, to estimate long-term shelter capacities, and to assess the damage to services that depend on essential infrastructure (e.g. hospitals, schools, etc.). Remote sensing techniques, including satellite imagery, can be used to gathering such information so that the overall damage can be assessed. However, specific points of interest among the damaged buildings need higher resolution images and detailed information to assess the damage situation. These areas can be further assessed through unmanned aerial vehicles and 3D model reconstruction. This paper presents a multi-UAV coverage path planning method for the 3D reconstruction of postdisaster damaged buildings. The methodology has been implemented in NetLogo3D, a multi-agent model environment, and tested in a virtual built environment in Unity3D. The proposed method generates camera location points surrounding targeted damaged buildings. These camera location points are filtered to avoid collision and then sorted using the K-means or the Fuzzy C-means methods. After clustering camera location points and allocating these to each UAV unit, a route optimization process is conducted as a multiple traveling salesman problem. Final corrections are made to paths to avoid obstacles and give a resulting path for each UAV that balances the flight distance and time. The paper presents the details of the model and methodologies, and an examination of the texture resolution obtained from the proposed method and the conventional overhead flight with the nadir-looking method used in 3D mappings. The algorithm outperforms the conventional method in terms of the quality of the generated 3D model.

## Introduction

When a disaster such as an earthquake or tsunami strikes, obtaining a comprehensive picture of the damage situation is one of the most important tasks in disaster response. For instance, to assess the extensive damage produced by the Great East Japan Earthquake of March 11, 2011, responders relied on aerial photographs and satellite remote sensing to avoid delays in information collection caused by the destabilization and limitations of ground transportation^1^. Especially when the accident at the Fukushima Daiichi Nuclear Power Plant occurred, satellite remote sensing and unmanned aerial vehicles (UAVs) were crucial platforms to avoid human exposure to radiation on the premises of the power plant^2^. Significant progress has been made in the estimation of building damage in disasters using satellite remote sensing^3, 4^. For instance, Moya et al. (2020)^5^ identified damaged areas based on change detection algorithms, while Moya et al. (2019)^6^ used three dimensional texture features and support vector machine (SVM) to detect collapsed buildings with high accuracy. The proposed method were tested using synthetic aperture radar data, and Lidar-based digital surface model (DSM). Thus, satellite remote sensing provides an initial estimation for constructing damage maps in wide areas; however, the details of the damage to buildings typically remain unclear because of the lack of information about the condition of the buildings’ walls and the satellite image resolution. For this reason, increasing attention is being paid to alternative airborne remote sensing systems, such as UAVs. Photos taken by UAVs can be used to construct a 3D model of a particular point of interest (POI) using the structure-from-motion (SfM) and multiview stereo (MVS) methods^7–9^.

Indeed, UAVs are currently being applied in several fields, such as aerial assessment of social infrastructure, aerial photography, aerial surveying, and pesticide spraying. In addition, UAVs are used for disaster site surveys because of their low cost and low measurement time requirements. Meanwhile, SfM technology has progressed by virtue of advances in computer vision. Currently, SfM techniques are able to extract many characteristic points from overlapping photographic images and simultaneously estimate the three-dimensional (3D) coordinates of elements and characteristic points of a camera image. Similarly, MVS generates high-precision 3D point groups or mesh models via a matching function among multiple photographic images based on the 3D geometric information collected through SfM. SfM and MVS are conducted simultaneously in most commercial software programs. Software tools such as Pix4D^10^ and PhotoScan^11^, among others, have become popular among UAV users. In fact, private companies and individuals can now conduct SfM surveys quite easily. However, in most commercial software packages, the mission planning feature is limited to a few standard configurations, such as grid missions with a nadir-looking camera (overhead flight) or a fly-around of a point of interest. The limited flexibility of these path planning algorithms means that several flights must be made to capture multiple targets in a complex environment^12^. This issue is critical in the case of disaster response, in which biases and a lack of information in surveys are of particular concern. Moreover, planning multiple flight missions and paths is a time-consuming process that can delay disaster relief activities. A precise 3D model of a complicated structure can be produced with the aid of commercial softwares. The method applied in these software packages combines the images acquired by the UAV with the images acquired on the ground^10^. A large body of work on 3D scanning, path planning and 3D reconstruction is available in the literature, here we briefly present the most salient work in 3D reconstruction and Coverage Path Planning (CPP) algorithms. Then, we developed a pipeline for multi-objective and multi-UAV path planning. Finally, the method is compared to the conventional stripmap nadir-looking flight pattern (hereafter, *the conventional approach*) used in 3D mapping of urban areas^13^.

In summary, we contribute the following: (i) a simple pipeline that allows users to allocate multiple targets and paths for a similar number of UAVs to reconstruct possible damaged buildings in 3D after a disaster; (ii) a general area division method based on the clustering and balanced task approach; (iii) a pipeline that ensures collision avoidance among UAV units and the environment; and (iv) a comparison of the 3D reconstruction quality of synthetic scenes of the proposed pipeline and the conventional approach.

### Motivation of the study

The authors’ research studies have been focused on exploring methodologies and alternatives to grasp the damage situation of buildings and essential infrastructure in the aftermath of a disaster. Satellite remote sensing technologies are used as the main tools to acquire the damage information within the impacted areas. We previously developed several methods to enhance building damage mapping by using pre- and post-disaster satellite imagery and change detection algorithms^5^, machine learning^14^ and deep learning methods as well^15^.

There is a great advantage in terms of the level of coverage of satellite images; however, there are limitations regarding the level of detail of the damage that can be grasped from a top-down view. In particular, there is concern regarding the condition of walls, which may have collapsed or have sustained structural damage and may compromise the stability of the building. Thus, to acquire such detailed information a closer look at the POI is necessary. In addition, to avoid the deployment of human teams that might incorporate new risks and demand additional costs and time, UAVs are a feasible alternative for this task. Thus, one of the motivations for this study is to tackle the limitations posed by satellite remote sensing damage estimation by integrating the advantages of UAV reconnaissance capabilities within the damage mapping pipeline.

To this end, the need to speed up the acquisition of images from various damaged structures require the implementation of multiple UAVs into the surveying task. In addition, it is important to consider a suitable workflow for CPP that ensures safe flight and high quality 3D reconstruction of the POIs.

## State of the art

### 3D reconstruction using UAVs

Nex et al.^13^ showcases various 3D reconstruction models from UAV platforms applied to various fields of study. They show a typical pipeline and the approximate time taken to produce a typical UAV-based photogrammetric workflow. It is suggested that “flight planning” takes only 5% of the whole workflow time, while “image acquisition” (20%) and “Digital Surface Model generation” (25%) are the tasks requiring the highest effort time. Thus, it may seem that the task of interest in this study is not significant; however, the estimations above refer to small areas and are based on the flight-planning of one UAV unit following a conventional approach. On the other hand, for wide areas, when increasing the number of UAV units, the complexity of flight-planning increases, thus the necessary time for flight planning might become significant in the workflow^16^.

In addition, Zheng et al.^17, 18^ developed a multi-UAV route planning methodology for 3D building model reconstruction. They focused on the use of multi-UAV for the reconstruction of a single building and used an initial model based on information extruded from the building footprint data of the POI. The created volumetric is then use to estimate the drone location to obtain sufficient coverage of the building. The idea is extendable to multiple POIs, and in the case of postdisaster environments, based on the damage mapping conducted from satellite remote sensing, as presented in the previous section, the footprint information can be updated to produce a more reliable initial map. Notice that at this point we are clearly ignoring other features and infrastructure in the urban environment that might pose a collision risk for drones (e.g. poles, electric cables, antennas, ad billboards, etc.). In addition, Hepp et al.^12^ proposed trajectory optimization to obtain high-quality 3D reconstruction of large buildings. First, a low-precision model of the area is generated using an overhead flight pattern. Then, they suggest an optimization of viewpoints to obtain the final path that will generate a suitable camera position constellation for sufficient coverage of the POI. A similar approach uses semantic segmentation from an initial flight to optimize the route and ensure a safe flight plan^19^.

### Coverage path planning

The problem of planning multiple tasks for multiple UAVs can be addressed as a ’divide-and-conquer’ approach^20^, in which the tasks are divided or allocated to each UAV unit and the subproblems are solved individually. In this case, the task is similar to a multiple traveling salesman problem (MTSP). Thus, first, a clustering of the visiting points (drone camera locations) is necessary. Jain et al.^21^ describes data clustering best practices, such as K-means. Previously, Barrientos et al.^22^ used a path planning algorithm to survey farms. They transformed the farm into a low-resolution, two-dimensional grid and then divided the area equally between the drone units. Next, a search algorithm with backtracking was used to find the paths that minimize the number of turns. Similarly, Bouzid et al.^23^ applied rapidly exploring random tree star fixed nodes (RRT*-FN) combined with the genetic algorithm (GA) by assuming that the CPP issue emulates a vehicle routing problem (VRP). In addition, Gao et al.^24^ used spanning tree coverage (STC) with ant colony optimization (ACO). On the other hand, Ju et al.^25^ applied a distributed swarm control algorithm to show that the performance of the multi-UAV system is significantly superior to the single-UAV system. They considered the following seven performance metrics: total time, setup time, flight time, battery consumption, inaccuracy of land, haptic control effort, and coverage ratio. Similarly, Ge et al.^26^ studied path planing for oilfield inspection within a 3D environment. Here, the cost function includes metrics such as: distance, height, time and electricity consumption. They proposed a combined use of metaphor-inspired metaheuristic algorithms like pigeon-inspired optimization (PIO) and fruit fly optimization algorithm (FOA). FOA is applied to account for dynamic obstacles and to increase computation speed. In recent years, Cabreira et al.^27^ presented a survey of studies focusing on CPP approaches in UAVs. Unfortunately, the CPP problem presented in their review is focused on 2D mapping representations where cellular decomposition is common. Such methods are conventionally used for area mapping; however, their resulting quality is limited by the flight pattern, which is basically a back-and-forth flight along parallel lines while keeping the camera position on a nadir looking angle.

Various CPP methods have been tested when clustering is applied. For instance, Zhao et al.^28^ studied task clustering and planning using K-means and simulated annealing (SA) to ensure balance in the task assignment. Later, Botteghi et al.^29^ confirmed that K-means performs well when the priority is to balance the distance and time among UAVs. Similarly, Mansouri et al.^30^ applied K-means as the clustering method for a collaborative CPP focused on single complex 3D infrastructure. Furthermore, Kong et al.^31^ proposed a fusion of the K-means and Dijkstra algorithms to allocate offline stores for sale staff and optimize the visiting path. This is a traveling salesman problem (TSP) solved by using clustering and shortest path calculations. The algorithm can evenly distribute the workload to differently sales staff. In our case, if a sales staff member is understood to be a UAV unit and an offline store is a camera location, then the algorithm can be transferred to our problem. However since points are in space, special care is needed when applying shortest path because trajectories may cross physical objects.

In addition, Bouras et al.^32^ developed a two-scale algorithm to plan a fleet of UAVs visiting various points of interest to scan an area for 2D ortho mapping. Similar to Bouzid et al.^23^, they set the CPP task assuming a VRP solved with SA. Here, UAV units consider a hovering time to take measurements and the flight is set to go to the POI and back to the station. Since their purpose is to map the area of interest without paying attention to specific POIs, these are not given and are calculated from an iterative process of voronoi tessellation and centroid selections. On the other hand, similar to the case we will present here, Xiao et al.^33^ focused on the CPP problem considering that the 3D reconstruction will be generated by an available software program. They focused on ensuring image overlap and reducing energy consumption. In contrast, they assume flight with a nadir angle and splitting the task of one model building among the available UAV units.

Furthermore, among recent studies, Ali et al.^34^ proposed a self-organization, collective motion and control of a UAV swarm. They used Particle Swarm Optimization (PSO) and Multi-agent System (MAS) to design an algorithm towards self-synchronization among UAVs. Similar to this study, they handle the problem in a 3D space and the verification of their method was conducted on a simulation-based approach. PSO, is one of the most used optimization algorithms in swarm research, in this regard, Shao et al.^35^ improved the PSO algorithm by introducing a chaos-based particle initialization method and adaptive parameter adjustments to improve optimallity and rapidity of the algorithm. The improvement proved to outperformed other similar PSO methods. In contrast to our study, Shao et al.^35^ focuses on simple trajectory with start-to-end points without intermediate goals, however similar to our case, they also used a simulated-based verification of their method.

In summary, clustering methods, such as K-means, have been applied to the CPP of multiple UAVs and solved for sub-trajectories as TSP or VRP problems using optimization algorithms. Unfortunately, most scholars applied this to a single structure with multiple UAVs. In contrast, we will explore the application of multiple structures with multiple-UAVs since a damage assessment requires the inspection of multiple targets.

### UAV for disaster mapping

Disaster situation awareness can be significantly improved by using UAV imagery. UAVs can be small, light-weight and easily deployed quickly. Thus, search and rescue, damage mapping and recovery monitoring are some of the tasks suitable for UAVs to perform. Moreover, UAV imagery has higher resolution than satellite and airborne photos; these are a significant data-set to map building damage^7^. For instance, Nex et al.^7^ focused on real-time damage mapping using UAVs. However, the flight plan is proposed for one UAV unit and with the purpose of exploring an unknown area. The photos obtained are used to detect damage through machine learning algorithms. In contrast, in this paper, we focus on a second stage of damage mapping, where essential infrastructure require greater details for assessment and a 3D reconstruction of each building is necessary. In this case a strip-map with the nadir-looking angle is not sufficient to obtain a detailed 3D reconstruction. Previous research has focused on 3D reconstruction of damaged buildings by using commercial off-the-shelf UAV units^36^.

## Methodology

**Figure 1:**
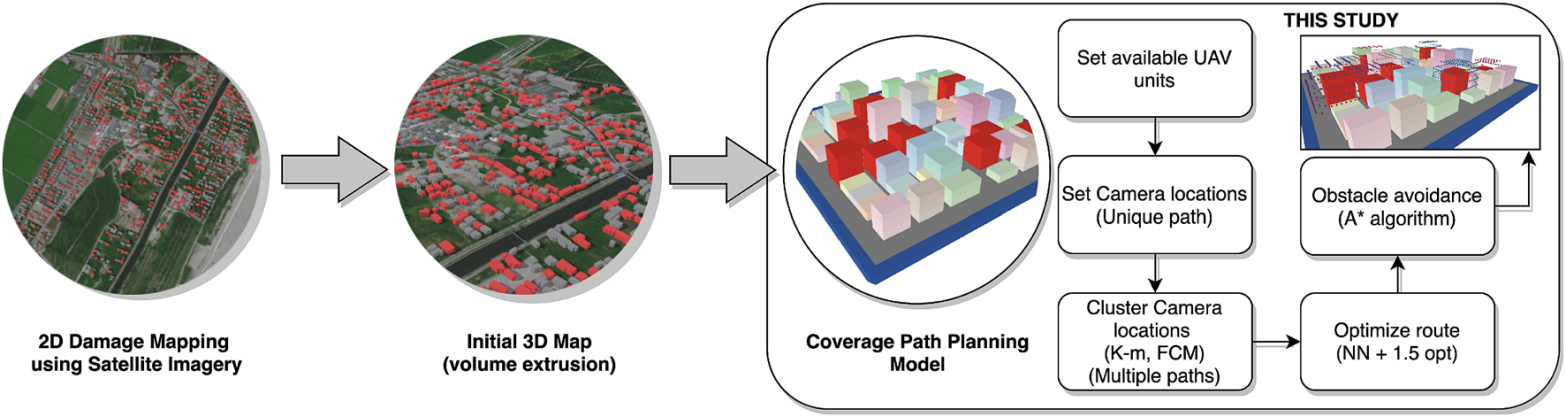
Workflow and scope of this study. Satellite remote sensing generates a 2D damage map. Volume extrusion from building geometries provides an initial 3D map, and the current model calculates the paths for each available UAV unit. *Source*: This study. The maps and virtual environment were developed in ArcGIS Pro and NetLogo3D, respectively.

We summarized the workflow in Fig. 1. Based on the information obtained from building damage mapping using satellite remote sensing an initial 3D map is constructed based on the extrusion of building geometries, as suggested in Botteghi et al.^29^. Another alternative would be to obtain the initial map through a low resolution and real-time reconstruction to, then, proceed to the CPP model. The CPP model will be the focus of this paper. To inspect all damaged buildings in the area, the necessary camera locations are generated as if a unique path is required (“Generation of camera location points” section). Next, based on the number of available UAVs (user setting), a cluster algorithm (i.e., *K*-means or Fuzzy *C*-means) is executed to calculate one path per UAV (“Clustering camera location points” section). The clustered points are joined to generate a unique path per UAV using the nearest neighbor (NN) and are improved with the 1.5-opt approximation algorithm (“Route optimization” section). Final routes are verified for collision avoidance and are corrected using the A* algorithm to overcome obstacles (“Obstacle avoidance” section).

**Figure 2:**
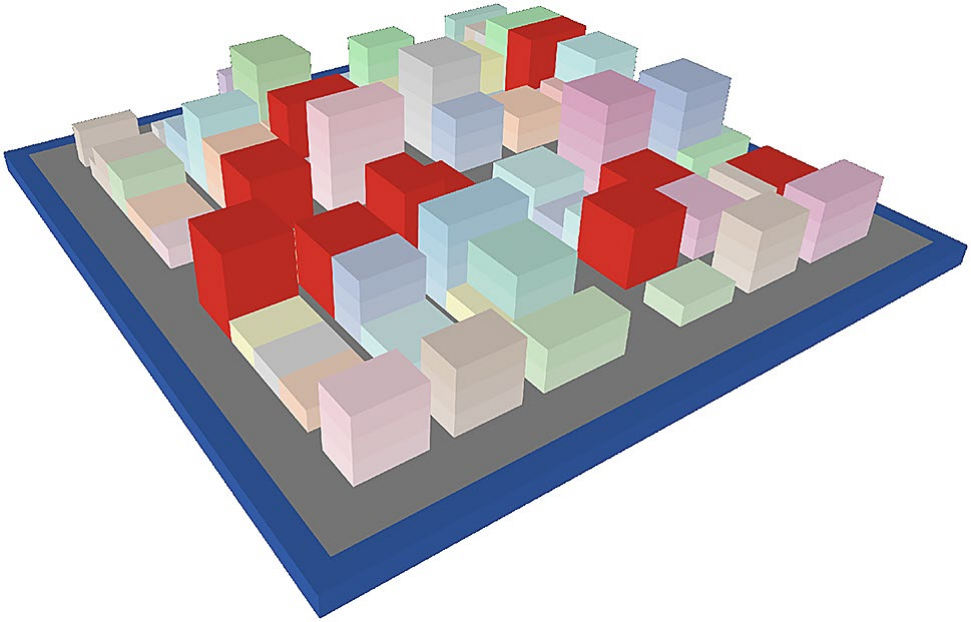
Virtual urban environment created in NetLogo3D. Here, the red volumes represent damaged buildings in need of inspection. *Source*: This study.

For the purpose of evaluating the method, we built a virtual urban environment using NetLogo3D^37^ (Fig. 2). A demo video can be found in the supplementary data. The configuration is set randomly and the structures to be surveyed are visualized in red. Here, the camera locations and flight paths are calculated.

### Generation of camera location points

First, camera locations separated every 2.5 m horizontally and 5 m vertically were generated around the objects of interest. The camera was presumed to face the center of the nearest object. In addition, locations satisfying any of the following conditions were deleted from the candidate list: (i) the distance to the object of interest is less than a certain user-defined value (7.5 m in this case); (ii) a structure exists between the object and the camera; and (iii) the coordinates of the candidate are inside of a structure. We selected a camera visibility angle of $90^{\circ }$ horizontally; additionally, the camera aspect ratio was set to 4:3, and the distance between the camera and the object was set to 7.5 m. Moreover, the images taken at each location were required to have a forward overlap ratio of at least 80% and a side overlap ratio of over 50% to ensure sufficient multiplicity.

### Clustering camera location points

Since the generated candidates for camera locations are shared among all UAVs, a clustering method for allocating specific locations to specific UAV units is necessary. Clustering is among the most studied problems in machine learning. Methods like SVM or Deep Learning models require training data, however the *K*-means method does not rely on training data.

#### K-means

The K-means method is an algorithm for non-hierarchical clustering. Clustering is the process of grouping a series of data. As a type of unsupervised learning method, non-hierarchical clustering is a method for directly classifying similar data into groups using an evaluation function. The *K*-means method follows a simple algorithm; therefore, it is a typical clustering method that is widely used^21^. Consider a set of camera locations $x_i$ ($i = 1,\ldots , n$) to be partitioned in *k* sets $S_j$ ($j = 1,\ldots , k$), where each set is to be allocated to a unique UAV unit. First, a random partition is used to initialize the algorithm by allocating the data into one cluster, thus, creating *k* clusters. Then, we find the centroids $m_j$ ($j = 1,\ldots , k$) of each cluster and update it as the new mean. The distance between each observation $x_i$ and each centroid $m_j$ is calculated to re-sort the data into their closest clusters.

The assignment and update of means can be represented as Eqs. (1) and (2), where *t* denotes the step which continues until convergence.

1 $$\begin{aligned} S_j^{(t)}= & {} \{x_i:\Vert x_i-m_i^{(t)}\Vert ^2 \le \Vert x_i-m_j^{(t)}\Vert ^2,\forall i, 1\le i \le n,\forall j,1\le j \le k\} \end{aligned}$$

2 $$\begin{aligned} m_j^{(t+1)}= & {} \frac{1}{|S_j^{(t)}|}\sum _{i=1}^n x_i \end{aligned}$$

Next, if the change in $m_j$ is less than a predetermined threshold value, the operation is terminated, otherwise a new assignment of clusters is conducted until convergence occurs.

In mathematical terms, the process described above is applied to minimize the following objective function:

3 $$\begin{aligned} {{\,\mathrm{\mathrm{argmin}}\,}}_S \sum _{j=1}^k \sum _{i = 1}^n \Vert x_i - m_j \Vert ^2 \end{aligned}$$

The performance of this method is known to strongly depend on the positions of the initial clusters. Therefore, the first application of this method might not yield the best sorting results. In addition, it is assumed that the clusters are spherical and that the number of clusters into which the data should be classified is known, thus classification is difficult if these assumptions are violated.

#### Fuzzy C-means

When each observation $x_i$ is sorted into just one cluster, such clustering is referred to as hard clustering. By contrast, when data are partially sorted into multiple clusters, such ambiguous clustering is known as a fuzzy clustering. The *K*-means method belongs to the former type of clustering, while the fuzzy *C*-means (FCM) method belongs to the latter. The FCM method provides each $x_i$ with an attribution degree $w_i$ ($0 \le w \le 1$) with respect to each cluster based on the distance to the centroid $c_j$.

The algorithm starts at $t=0$ with a random assignment of attribution degree coefficients. While there is no convergence (the change in coefficients’ values between two iterations is lower than a threshold), the algorithm loops on computing the centroid of each cluster $c_j^{(t)}$ ($1 \le j \le c$). In the FCM method the centroid is the weighted mean of the camera location points with the degree of attribution to the cluster as the weight [Eq. (4)]. In addition, a hyper-parameter *m* is defined to control the *fuzziness* of clusters. Next, the partition matrix or attribution degree matrix $w_{ij}$ is calculated from Eq. (5).

4 $$\begin{aligned} c_j^{(t)}= & {} \frac{\displaystyle \sum \nolimits _{i = 1}^n {(w_{ji}^{(t - 1)})}^m x_i }{\displaystyle \sum \nolimits _{i = 1}^n {(w_{ji}^{(t - 1)})}^m } \end{aligned}$$

5 $$\begin{aligned} w_{ij}^{(t)}= & {} \dfrac{1}{\displaystyle \sum \nolimits _{k = 1}^c {\left( \dfrac{\Vert x_i-c_j^{(t - 1)}\Vert }{\Vert x_i-c_k^{(t - 1)}\Vert }\right) }^{\dfrac{2}{(m - 1)}}} \end{aligned}$$

The value of *m* is usually set to a real number between 1.3 and 2. In the limit $m = 1$, this method reduces to the *K*-means method of hard clustering. In mathematical terms, this method aims to minimize the following objective function:

6 $$\begin{aligned} {{\,\mathrm{\mathrm{argmin}}\,}}_C \sum _{i = 1}^n \sum _{j = 1}^{c} {(w_{ij})}^m \Vert x_i - c_j \Vert ^2 \end{aligned}$$

### Route optimization

With camera locations clustered and sorted to each UAV unit, the next step is to create optimum paths for each UAV within its clustered points. Then, the multiple traveling salesman problem (MTSP) is solved in this study. Here, each UAV visits all camera location points in its cluster only once and then returns to the starting point. For all UAVs, the starting point and the end point after the flight were assumed to be the same based on practical considerations. The MTSP for the UAV routes was solved to select one solution for which the cost was minimal and uniform across all devices.

**Figure 3:**
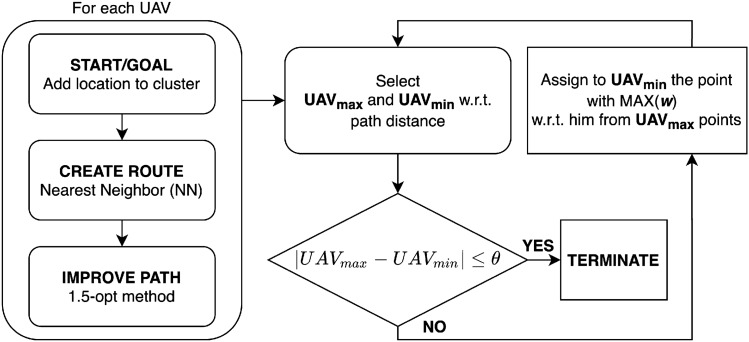
Process to calculate optimum paths for UAVs. *Source*: This study.

The UAV routes are created using the nearest neighbor (NN) method and are improved with the 1.5-opt algorithm (hereafter *NN method*). Next, we select the UAVs with maximum and minimum distance cost ($UAV_{max}$ and $UAV_{min}$ in Fig. 3) using Eqs. (7) or (8) when optimizing by distance or flight time, respectively. When the difference in the distance costs exceeds a given threshold ($\theta$), the camera locations of $UAV_{max}$ are inspected. Among them, the maximum attribution degree point w.r.t $UAV_{min}$’ cluster is re-assigned to $UAV_{min}$.

7 $$\begin{aligned} f_i= & {} \sum _{k=1}^{n} d_k \end{aligned}$$

8 $$\begin{aligned} f_i= & {} t_h (n - 1) + \sum _{k = 1}^{n} t_k \end{aligned}$$

9 $$\begin{aligned} t_k= & {} \left\{ \begin{array}{ll} \displaystyle \frac{d_k}{v_{\max }} + \frac{v_{\max }}{a} &{} \left( d_k> \displaystyle \frac{{v_{\max }}^2}{a}\right) \\ \displaystyle 2 \sqrt{\frac{d_k}{a}} &{} \left( d_k > \displaystyle \frac{{v_{\max }}^2}{a}\right) \end{array} \right. \end{aligned}$$

where $f_i$ is the cost function in step *i*; notice that this can be the flight distance or flight time. When optimizing by flight distance, $d_k$ denotes the distance between camera locations *k* and $k+1$. When optimizing by flight time, $t_k$ denotes the flight time between camera locations *k* and $k+1$, and $t_h$ denotes the hovering time at a point of acquisition. In both cases, *n* is the total number of acquisition points. Since each UAV is presumed to accelerate to its maximum speed with uniform acceleration and to hover at the camera location, $t_k$ can be calculated as Eq. (9), where $v_{max}$ is the maximum speed of a UAV, and *a* represents the uniform acceleration of a UAV.

### Obstacle avoidance

The routes found through the procedure presented in the preceding subsections may pass through obstacles when connecting the home location to the first camera location of each path. Thus, the $A^{*}~algorithm$ was implemented for obstacle avoidance in the cases where such a situation is present. We selected the $A^{*}~algorithm$ for finding a path between two points because it is more efficient than the Dijkstra method. Therefore, when two connected points of a path cross an obstacle, adjacent intermediate points are generated between these two and an alternative route is constructed for that segment.

**Figure 4:**
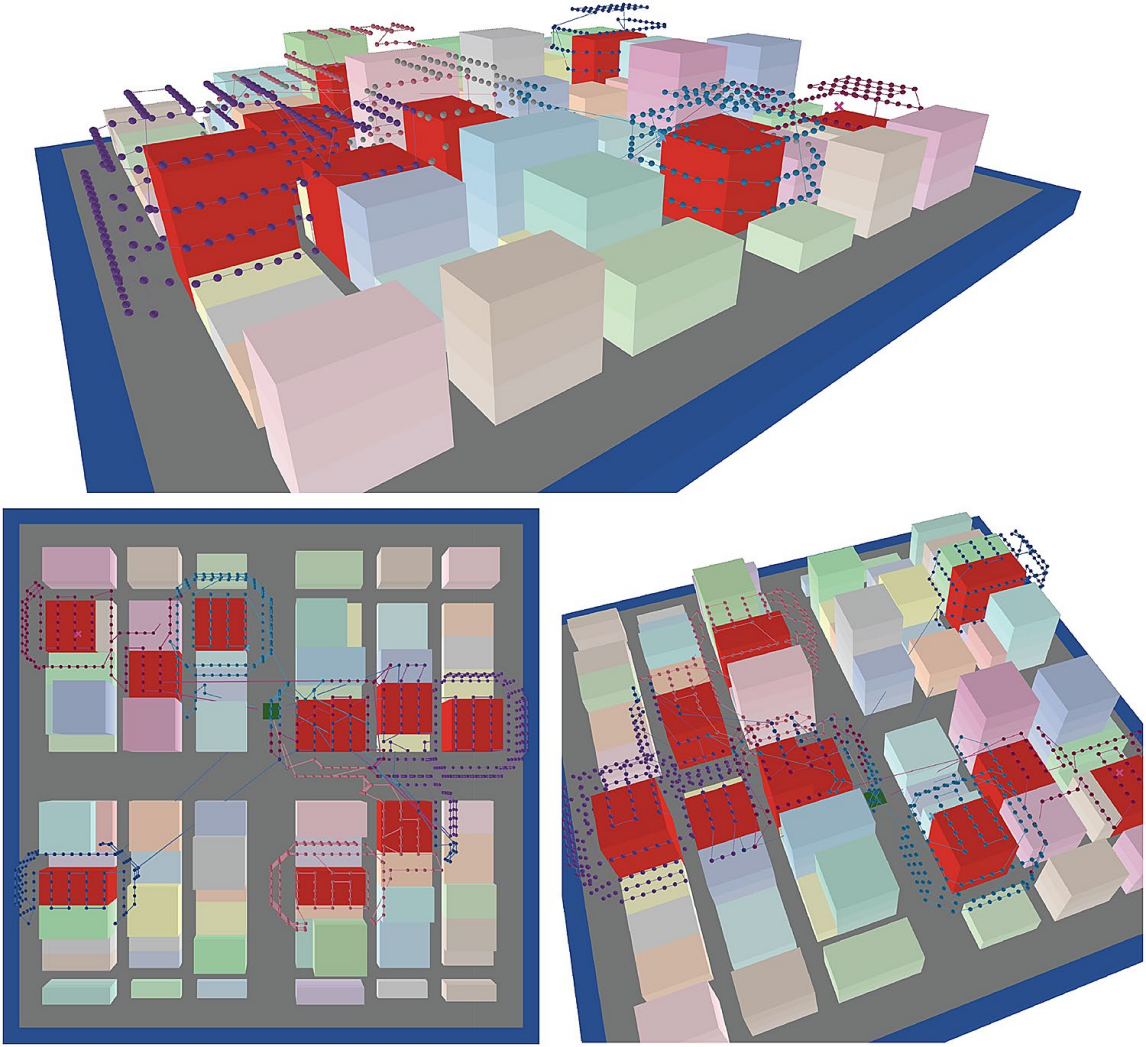
Final UAV routes produced using the proposed method. The green square at the center of the area indicates the home point for the UAVs. *Source*: This study, using NetLogo3D.

Finally, through the steps described above, the CPP for multiple UAVs is generated. Figure 4 presents an example of the output of the UAV flight routes of our example case of a virtual urban environment. Each point represents a camera location, and the edge color corresponds to the particular UAV to which that point is assigned. The points are linked by edges in accordance with the flight progression.

## Model-based analysis

### Analysis of clustering methods

To confirm the benefits of the FCM sorting method used in the proposed approach, it was compared to the *K*-means method. The environment shown in Fig. 2 was used for the comparative analysis. Five UAVs were used to survey 10 randomly selected structures at the site. After camera location points were generated for each structure, these were clustered using the *K*-means and FCM, optimizing the distance or flight time as explained in “Obstacle avoidance” section. In the *K*-means clustering case, the camera location points did not have attribution degree matrices, therefore, the cost of the TSP was minimized instead for each UAV. After a 100 trials of the above-described procedures, the results were examined for comparison. The cost functions provided in both Eqs. (7) and (8) were considered. The parameters were as follows: the constant *m* in the FCM method was set to 2, the UAV hovering time $t_h$ was assumed to be 2*s*, the maximum UAV speed $v_{max}$ was 5*m*/*s*, and the uniform UAV acceleration *a* was $2 m/s^2$. The total cost *F* was given as shown in Eq. (10). In addition, as another measure to evaluate clustering methods, the subtour averaging coefficient $\rho$ is considered, as shown in Eq. (11).

10 $$\begin{aligned} F= & {} \sum _{i=1}^{5} f_i \end{aligned}$$

11 $$\begin{aligned} \rho= & {} \displaystyle \frac{\max f_i - \min f_i}{\max f_i} \end{aligned}$$

**Figure 5:**
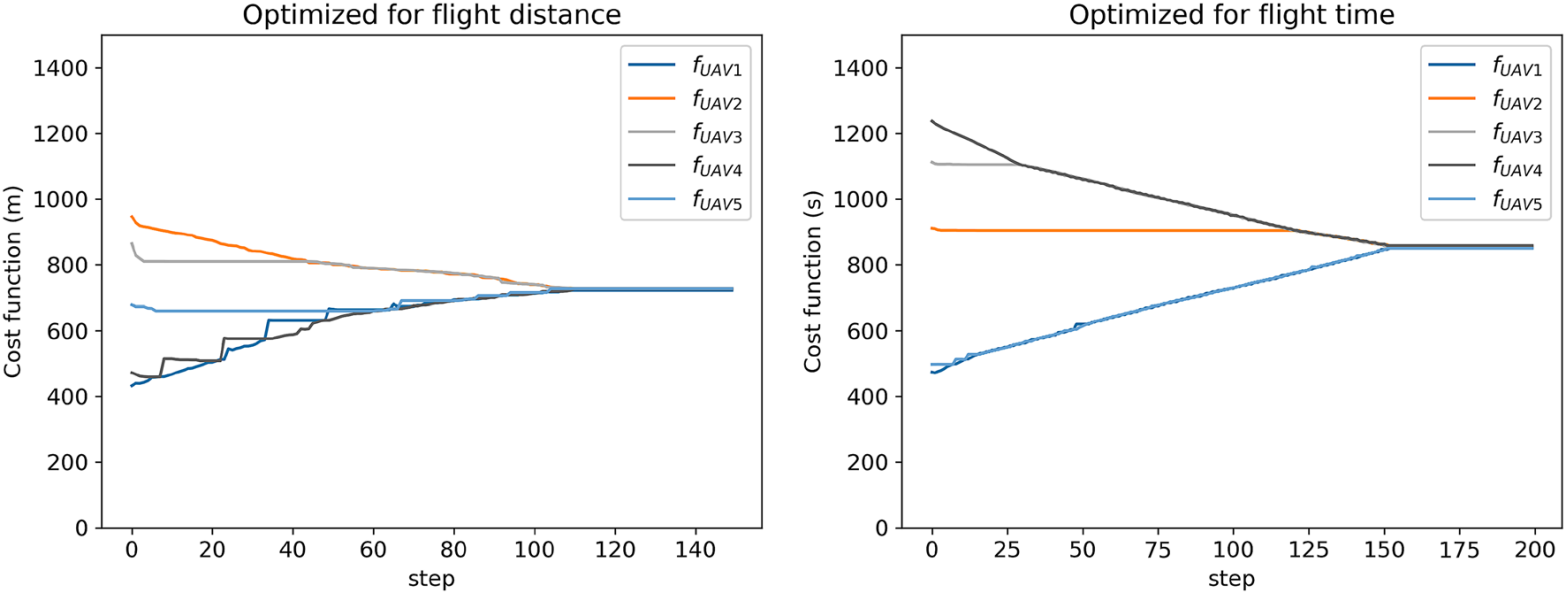
Changes in the cost functions $f_i$ given by Eq. (7) (left) and Eq. (8) (right) with the redistribution of camera location points based on the degree of attribution.

Figure 5 shows the cost functions for each UAV in the FCM method. Figure 5—left shows the cost function calculated using Eq. (7), whereas Fig. 5—right shows the results with Eq. (8). These results suggest that the redistribution of the camera location points among the UAVs based on the attribution degree function of the FCM method drives the cost functions for all UAVs to approach the average value. Tables 1 and 2 present the average values of the cost functions given by Eqs. (7) and (8), respectively. These tables show that the FCM method yielded a smaller subtour averaging coefficient and more uniform solutions, whereas the *K*-means method found a slightly lower total cost. One reason for this result is that with the hard clustering of the *K*-means method, long paths between structures might not be generated. Another is that $f_{min}$ increased more rapidly than $f_{max}$ decreased with redistribution based on the attribution degree, as shown in Fig. 5. However, the $\max f_i$ column in Table 2 shows that the *K*-means method resulted in a cost of approximately 20 *min* on average, whereas redistribution with the FCM method reduced the cost to less than 15 *min*. The current capacity limit of UAV batteries is typically 10–30 *min*, depending on the equipment. The termination of UAV operation is determined by the UAV with the maximum cost. Therefore, it is better to have a smaller maximum cost. From the discussions presented above, we can conclude that the clustering of the camera location points in accordance with the FCM method is quite effective in making the costs for multiple UAVs minimal and uniform when solving the MTSP.

**Figure 6:**
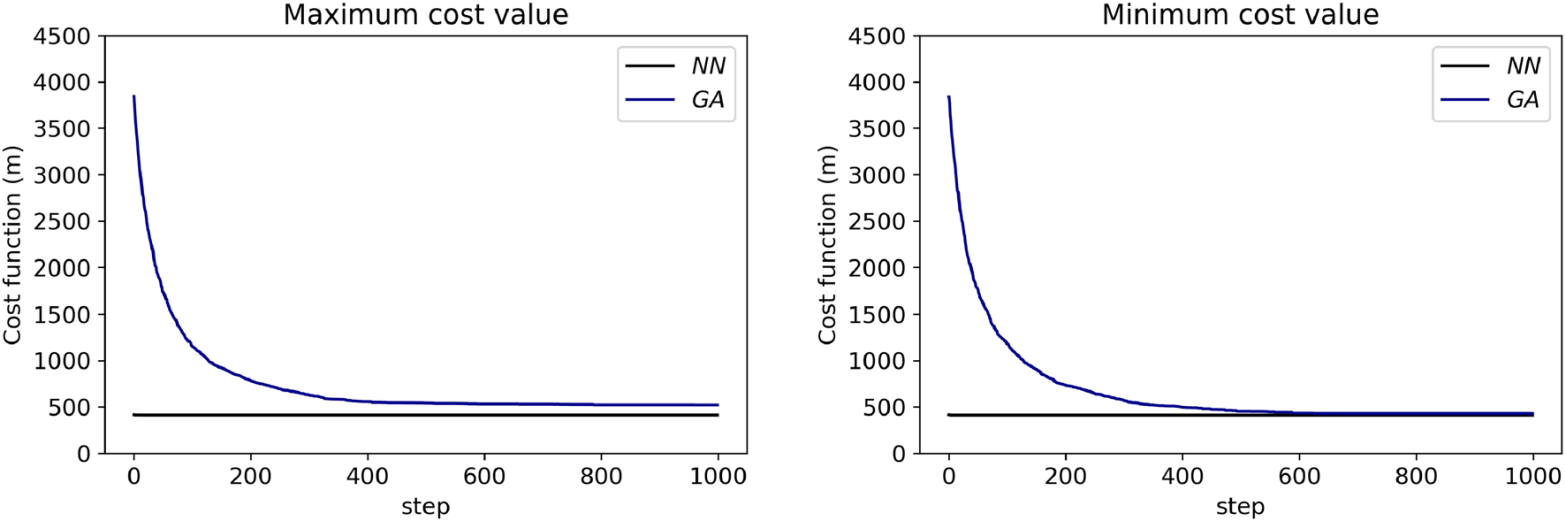
Convergence processes of the NN and GA methods resulting in convergence to the maximum cost value (left) and the minimum cost value (right).

**Table 1:** Average values of evaluation functions when the flight distance is used as the cost function.

	${\varvec{F}}$	${\varvec{\rho }}$	$\max f_i$	$\min f_i$
*K*-means	3236	0.545	902	405
Fuzzy *C*-means	3633	0.022	735	719

**Table 2:** Average values of the evaluation functions when the flight time is used as the cost function.

	${\varvec{F}}$	${\varvec{\rho }}$	$\max f_i$	$\min f_i$
*K*-means	4167	0.626	1218	455
Fuzzy *C*-means	4275	0.015	861	847

### Analysis of the route optimization method

To examine the effectiveness of the proposed workflow including the NN method, the precision of the path solution is compared with that achieved using the genetic algorithm (GA). We use the same virtual environment presented in Fig. 2. The GA parameters used here are presented in Table 3. Because both methods use random numbers, to ensure a fair comparison, 100 trials were conducted using both methods. The cost function given in Eq. (7) was used here.

**Table 3:** Parameters of the GA.

Parameter	Value
Crossover design	One-point crossover
Number of genomes	300
Tournament rate	1%
Mutation rate	10%

The results are presented in Table 4. Figure 6 illustrates the convergence of the cost function for both methods. Figure 6—left shows the case with the maximum converged value of the cost function among the 100 trials for each method, whereas Figure 6—right shows the case with the minimum converged value. Table 4 shows that the NN method produced more precise results. Basically, the time for one step is shorter in the 1.5-opt method than in the GA method. Ultimately, the method proposed in this study is approximately five times faster than the GA method. Figure 6—right demonstrates that even the initial solution provided by the NN method is more precise than the final solution of the GA method. Moreover, with the 1.5-opt method, convergence was attained in 20 steps, whereas the GA method took 600 steps to reach convergence. Therefore, the combination of the NN and 1.5-opt methods is superior to the GA method in terms of both precision and convergence speed. The reason might be that the combination of the NN and 1.5-opt methods may be particularly well suited to the current problem under examination, in which the camera location points are distributed regularly but with some deviations. A method in which the initial solution is obtained via the NN method but the subsequent improvement is achieved using the GA method requires more time for convergence than the current method. Improving the initial solution using the 1.5-opt method might be more compatible with the NN method than improvement via the GA method.

**Table 4:** Precision of the TSP solutions obtained using the NN and GA methods.

	NN method	GA method
$\min f_i$	410.9	428.2
$\max f_i$	411.7	522.0
Processing time (s)	29.3	146.7

### Analysis of the 3D reconstruction output quality

**Figure 7:**
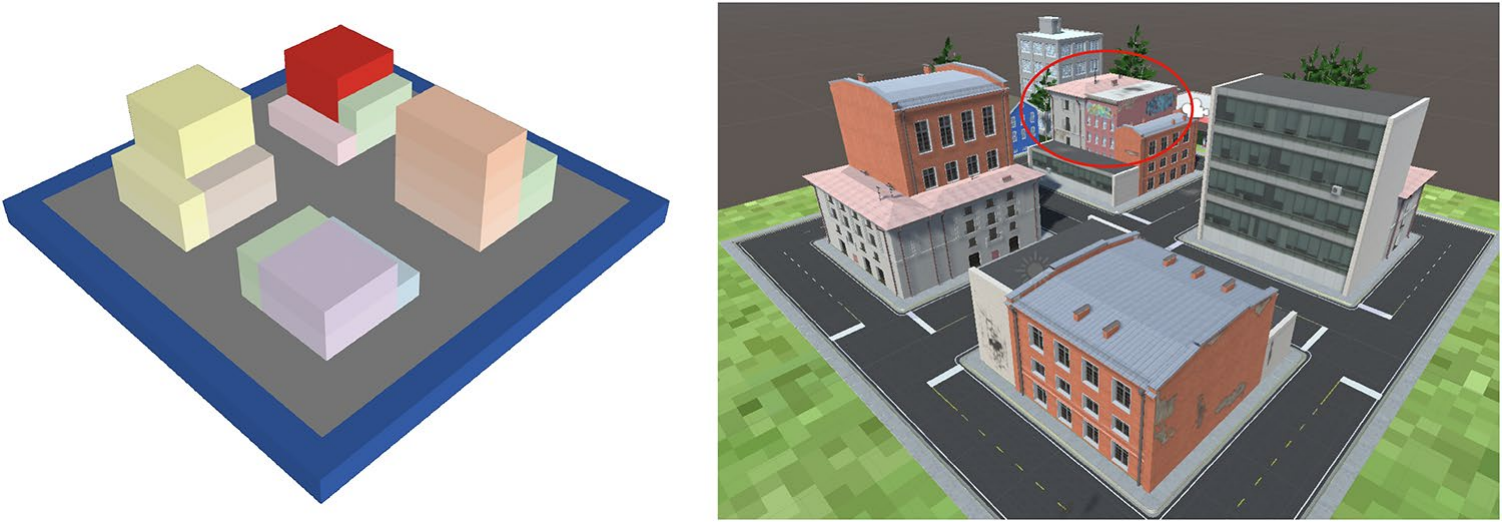
Left—Paths are generated to survey the red building from the NetLogo3D environment. Right—Blocks generated with Unity3D for quality assessment. *Source*: This study, using NetLogo3D and Unity 3D.

**Figure 8:**
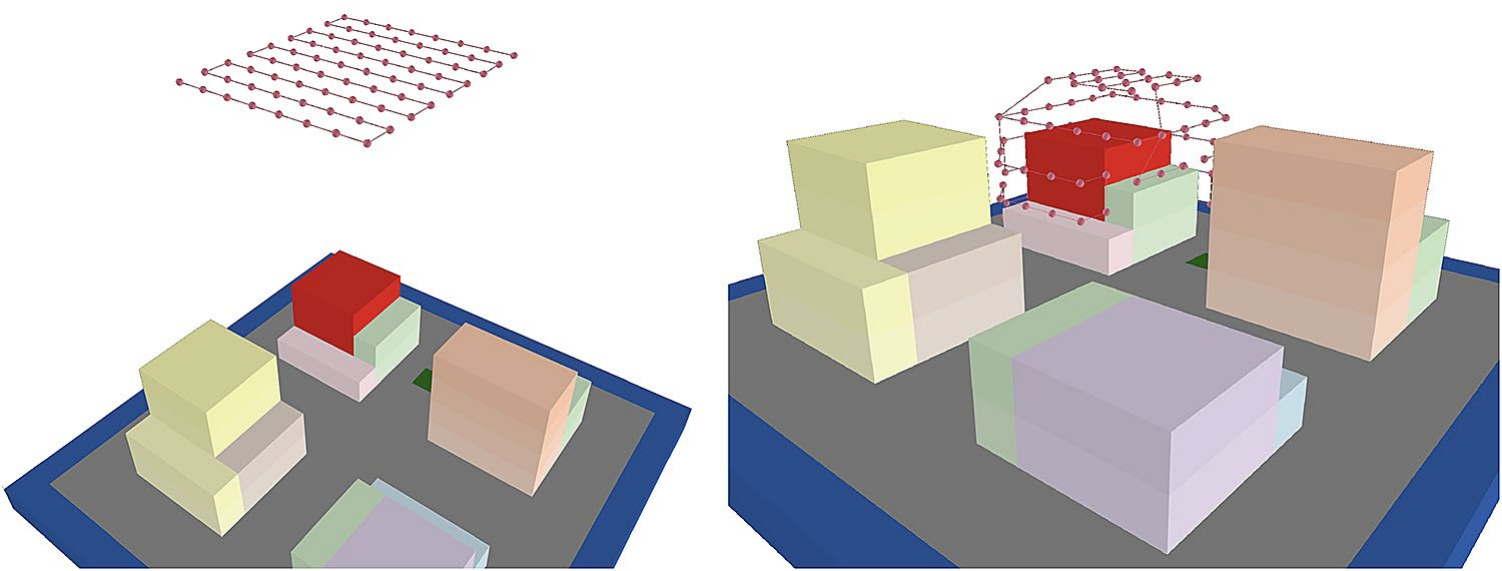
Left—Overhead flight route. Right—The path generated by this study. *Source*: This study, using NetLogo3D.

The proposed path generation method was examined in terms of its ability to enhance a more precise 3D model than that produced by a conventional overhead flight. We develop another virtual urban environment, as shown in Fig. 7, which was also generated using the Unity3D game engine^38^, as shown in the right panel of the same figure. The red oval indicates the object to be surveyed. A flight route for one UAV was designed using the proposed method, including the coordinates of the camera location points, the camera orientation, and the order of the camera location points to be visited. The routes are shown in Fig. 8

.

**Figure 9:**
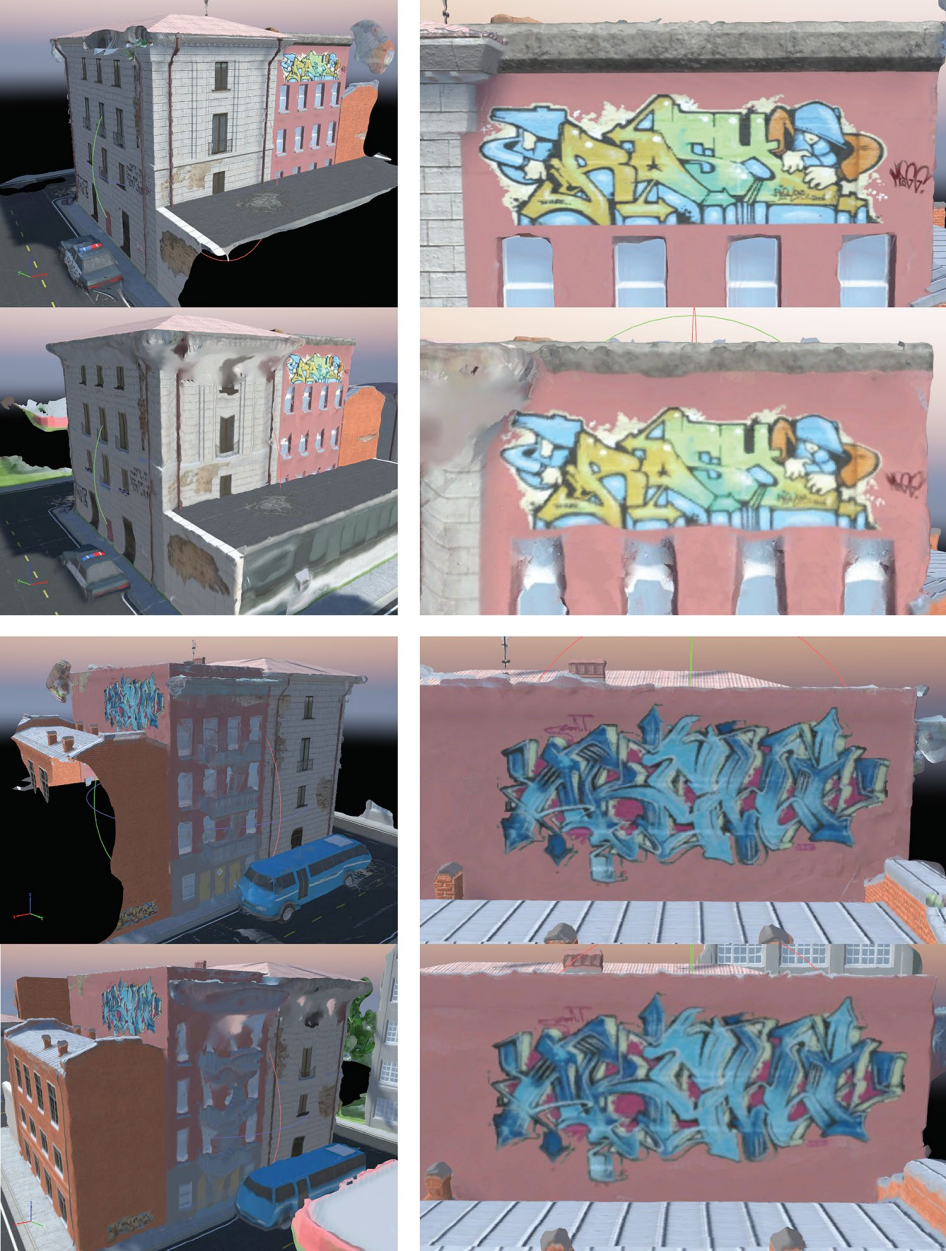
Comparison of two graffiti drawings. The top panel in each pair of images shows the results from the method proposed in this study, while the bottom panel shows the results of the overhead flight method. *Source*: This study, using Unity 3D.

**Figure 10:**
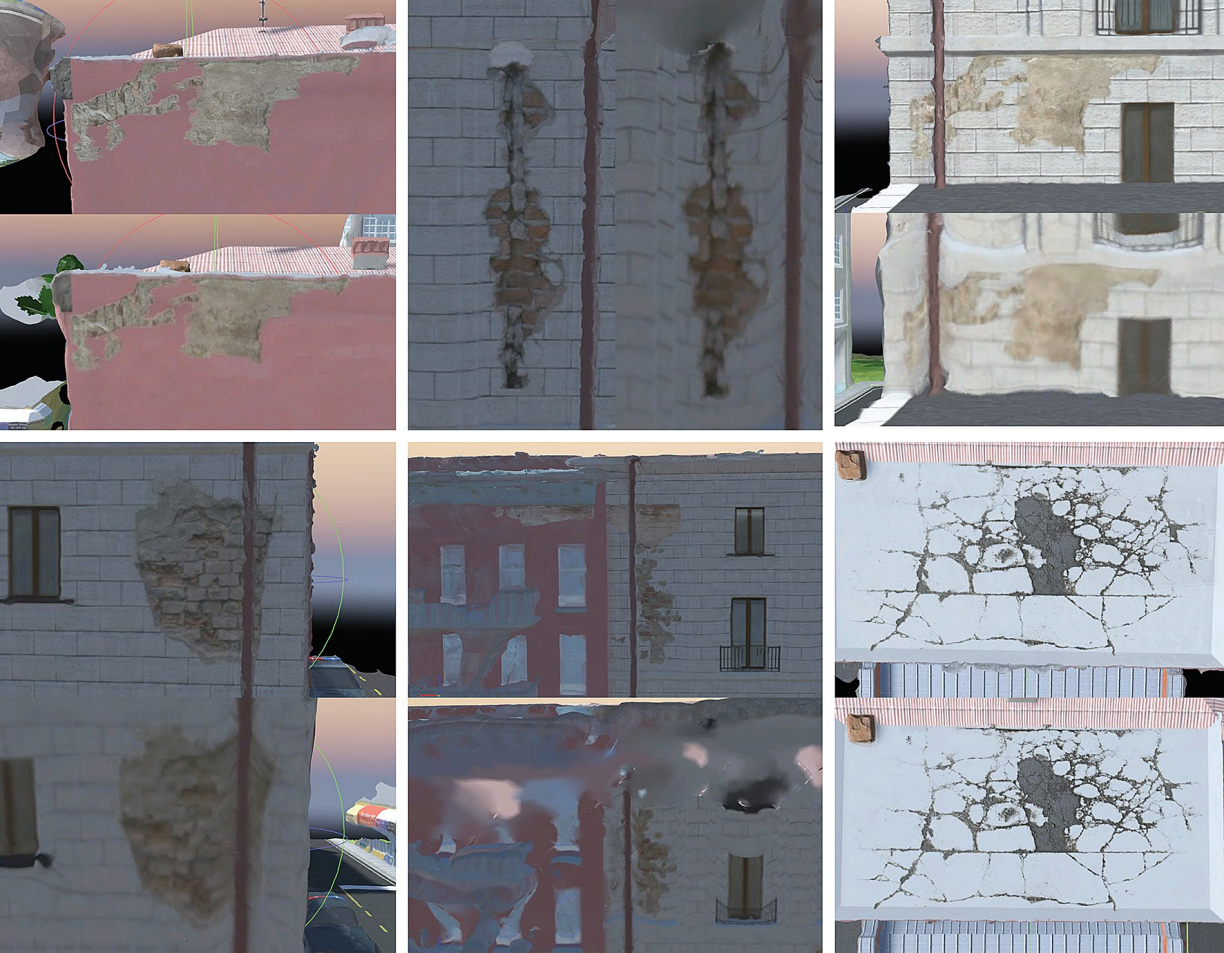
Comparison of the extraction of six damage features. The top or left panels in each pair of images shows the results from the method proposed in this study, while the bottom or right panel shows the results of the overhead flight method. *Source*: This study, using Unity 3D.

These flight routes were used in Unity3D to photograph the structure. The UAV took pictures at every camera location point on the flight path. The camera parameters are presented in Table 5. Jiang et al. (2020)^39^ evaluated the performance of six SfM software packages. Among them, Pix4D demonstrated the highest accuracy and, therefore, is used in this study to generate our 3D models for both flights, and their respective results are compared. Table 6 presents the parameters applied in Pix4D. In addition, as a comparative examination, the graffiti and damage details are illustrated in Figs. 9 and 10 to examine the precision of their reproduction.

**Table 5:** Camera parameters.

Parameter	Value
Visibility (vertical)	70
Size of sensor x	70
Size of sensor y	51
Focal length	36.418
Aspect ratio	4:3
Resolution	$1440 \times 1080$

**Table 6:** Parameters of Pix4D.

Parameter	Proposed method	Conventional method
Number of images	145	144
Quality	Standard	Standard
Coordinate system	Orthogonal	Orthogonal
Horizontal accuracy (m)	0.01	0.01
Vertical accuracy (m)	0.02	0.02

For the drawings of the walls shown in Fig. 9 both methods produced similar results, however, our results show better quality for the details, such as the window and the edge of the roof. This is expected since the overhead flight cannot acquire images of those spaces. Our results in the second case (Fig. 10) show even greater enhancement than the standard approach. Even on a shady side where only a few tie points could be extracted, the proposed method precisely reproduced cracks and graffiti.

## Discussion and future work

There is room for improvement of the proposed method, in particular when generating camera location points and redistributing those using the attribution degree. Regarding the former, the authors have proposed a rule-based camera location points generation method with sufficient overlap and sidelap ratios to achieve fast generation of camera location points. As a result of this method, erroneous extraction of tie points can occur. Additionally, a tendency toward very poor estimation of the camera position and orientation for short distances and multiple photographs is apparent. Therefore, the optimal photography points for the target objects must be generated after solving the network designing problem^12^. Regarding the redistribution of camera location points, the results show that the UAV costs when applying FCM are more uniform than those achieved by the *K*-means method. However, the *K*-means method is superior in terms of the total and average costs. In the examination performed above, the redistribution of the camera location points was performed for five UAVs. If camera location points are to be sorted among more UAVs, groups of clusters that are not mutually close will tend to appear, and long paths might be generated through redistribution using the same cost function and degree of attribution settings used in the present study. To avoid this pitfall, one might renew the centroid *m* of the clusters in the algorithm used for the MTSP by providing a weight coefficient ${\varvec{w}}_{{\varvec{i}}}$ for the Euclidean norm of the *K*-means method depending on the cost calculated for the MTSP. In this way, the method proposed herein could be extended to more UAVs, allowing it to yield better results in terms of the total and average costs compared to the method for solving the TSP for each UAV in the *K*-means clustering. Another shortcoming of the present proposal is the practical problem that it cannot respond quickly to a cost greater than the threshold cost. This shortcoming derives from the fact that a long computation time is necessary to achieve convergence of the UAV costs. Only in the final stage of processing can it be known whether the cost exceeds the threshold cost. To overcome this difficulty, one might predetermine the number of UAVs corresponding to the generated number of camera location points. However, if the converged cost value exceeds the threshold value, then the calculations must still be repeated for an increased number of UAVs.

A method that can uniformly minimize the costs for several tens of UAVs would be useful for developing methods for surveying much wider areas. The ability to provide highly precise 3D information about disasters over a wide area is expected to contribute greatly to the preparation of countermeasures to mitigate the effects of future disasters. In addition, the present study provides a model-based analysis using a virtual environment, however, real experiments should be performed in future work to further evaluate the application of the proposed workflow.

## Concluding remarks

We have proposed a path planning method for multiple UAVs to aid in 3D building damage surveys or disaster situations. The proposed methodology combines the fuzzy *C*-means method for assigning camera location points to each UAV with a route optimization algorithm for calculating the visit order of the camera location points for each UAV by solving the multiple traveling salesman problem. Finally, the selected paths are corrected to avoid obstacles between the camera location points using the A* algorithm. To assess the effectiveness of the proposed method, a comparative study of the quality of 3D reconstruction models was conducted, where the enhanced results confirm the superiority of our proposed approach over a conventional overhead flight mapping technique. We believe, the proposed method can improve reconnaissance capabilities in increasingly complex urban disasters.

## Supplementary Information

Supplementary Video 1.
